# Reply to “Re-evaluating the evidence for facilitation of stickleback speciation by admixture in the Lake Constance basin”

**DOI:** 10.1038/s41467-021-23096-x

**Published:** 2021-05-14

**Authors:** David A. Marques, Kay Lucek, Vitor C. Sousa, Laurent Excoffier, Ole Seehausen

**Affiliations:** 1 Aquatic Ecology and Evolution, Institute of Ecology and Evolution, University of Bern, Bern, Switzerland; 2 Department of Fish Ecology and Evolution, EAWAG Swiss Federal Institute of Aquatic Science and Technology, Center for Ecology, Evolution and Biogeochemistry, Kastanienbaum, Switzerland; 3 Computational and Molecular Population Genetics, Institute of Ecology and Evolution, University of Bern, Bern, Switzerland; 4 Department of Environmental Sciences, University of Basel, Basel, Switzerland; 5 Centre for Ecology, Evolution and Environmental Changes, University of Lisbon, Lisbon, Portugal; 6 Swiss Institute of Bioinformatics, Lausanne, Switzerland

**Keywords:** Evolutionary genetics, Adaptive radiation, Population genetics

**Replying to** Daniel Berner. *Nature Communications* 10.1038/s41467-021-23092-1 (2021)

A Matters Arising article^1^ raised concerns about the interpretation of our findings reported in our recent publication on admixture-facilitated ecological speciation in Lake Constance stickleback^2^. After careful consideration of the criticism, including additional analyses testing the proposed alternative hypotheses, we can confirm our confidence in the inference of secondary contact between a West European and an East European stickleback lineage in the catchment of Lake Constance, and that this admixture facilitated the ecological divergence between lake and stream ecotypes within Lake Constance^2^.

In particular, Berner^1^ (i) questioned whether West and East European stickleback populations should be considered as divergent lineages, (ii) suggested that Lake Constance stickleback originated from the upper Danube instead of East Europe, (iii) questioned the suitability of our demographic modelling approach to reject an ‘ecological vicariance’ scenario, (iv) proposed that divergent selection within Lake Constance biased our inference of a secondary contact and admixture scenario, and (v) criticized our conclusion on admixture-facilitation of ecological speciation as premature. We address each of these concerns in this sequence.

## Divergent West and East European lineages

**Fig. 1:**
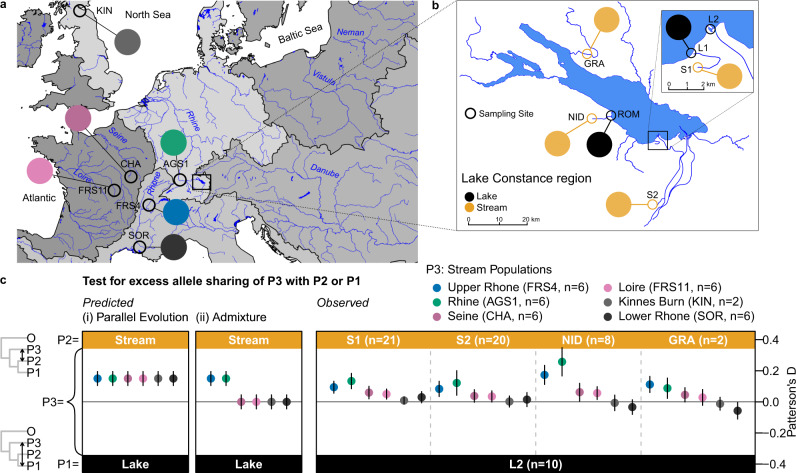
Signatures of secondary contact and admixture are not artefacts of parallel evolution. As uniquely predicted by the hypothesis of admixture between Rhine / upper Rhone and East European lineages in Lake Constance^2^, Constance stream stickleback show much stronger excess allele sharing with Rhine and upper Rhone populations than with other stream-adapted stickleback from West and South Europe. In contrast, a parallel evolution bias would predict excess allele sharing between all stream-adapted stickleback populations with Lake Constance stream stickleback, compared to the lake ecotype. **a** Map of European stickleback populations used in tests for excess allele sharing with colours indicating population identity, with (**b**) inset showing the Lake Constance region where colours indicate lake or stream habitat. **c** Predicted and observed patterns of excess allele sharing between West European stream-adapted stickleback (P3) and Lake Constance stream stickleback (P2), compared to Lake Constance lake stickleback (P1). Coloured dots show the estimate of Patterson’s D for each comparison and whiskers ±3 standard errors derived from a standard block-jackknife procedure. *Gasterosteus wheatlandi* (*n* = 1) was used as outgroup (O in the tree) in all tests for excess allele sharing, and all topologies are supported by P1 / P2 showing the highest shared derived allele count (number of “BBAA” patterns). Brackets next to population abbreviations give the number of individuals per population used in each test (e.g., *n* = 6 individuals for population FRS4). Watershed maps are derived from “Water Base: Global River Basins” by The World Bank used under CC BY 4.0, river and lake maps from “European catchments and Rivers network system (Ecrins)” by the European Environment Agency (EEA). Source data are provided as a Source Data file.

The deepest divergence among European threespine stickleback is between the Trans-Atlantic and South European clades^3–6^, with West and East European and Lake Constance stickleback part of the former, as we had clearly stated^2^. Within the Trans-Atlantic clade, hierarchical subclades exist that are structured by geography with divergence times estimated by others between 37 and 6.5 ky^6,7^, consistent with isolation in distinct glacial refugia^3^ or between different river catchments colonized during postglacial range expansion^6,7^. We referred to these as “divergent lineages”, consistent with our demographic modelling based estimates of ~8000 years between West (Rhine/upper Rhone) and East (Vistula) European populations^2^ (assuming 2 years generation time based on the average lifetime reproductive age rather than the age of first reproduction^8^) and high genomic differentiation (Fig. 5a in^2^). The limited bootstrap support for a reciprocally monophyletic West European stickleback clade in a new phylogenetic analysis of Berner^1^ (grey rectangle in Fig. 1 in^1^) is irrelevant to the argument and a consequence of the inclusion of hybrid populations (e.g., Lake Constance, upper Danube populations, see below) reducing internal branch bootstrap support^2,9^. The new analyses of Berner^1^ thus do not contradict our interpretation that stickleback populations from West and East European river catchments are old and divergent lineages^2^.

### Origin and timing of colonization

According to Berner^1^, phylogenetic clustering of Lake Constance stickleback with upper Danube stickleback suggests a natural colonization of Lake Constance through its postglacial connection to the Danube 15-10 ky ago^10^, as shown for other fish species with Danubian or mixed Danubian and Rhine ancestry and native to Lake Constance^11–13^. However, the history of hybrid lineages cannot be resolved on the sole basis of a phylogeny from concatenated markers. Evidently, the two individuals from the Lake Constance GRA population in Berner’s analysis cluster in two different clades (Fig. 1 in^1^, Supplementary Fig. 1d), which is consistent with our previously estimated 50:50 West vs. East European ancestry^2^. We previously assumed that upper Danube stickleback are hybrids from multiple introductions including West European lineages^14^ and thus excluded them from our initial analysis^2^. Now, we have tested this assumption: upper Danube stickleback indeed show an admixture signature between West and East European populations just like Lake Constance stickleback (Supplementary Fig. 2), including phylogenetic analyses clustering them with either West or East European lineages depending on the context of what other lineages are included in a tree (Supplementary Fig. 1e). An admixed West / East European origin of both Lake Constance and upper Danube stickleback thus explains their similarity and placement in a phylogeny dominated by hybrid populations (see^1^, Supplementary Fig. 1d).

When and how stickleback got into Lake Constance and into the upper Danube remains difficult to resolve with genomic data alone, considering the complexity of demographic models^2,15^ and uncertainties in underlying mutation rates. In particular, the inference of split times based on the site frequency spectrum (SFS) relies on a known mutation rate or a known effective size^16^. We used a gene alignment based^17^ mutation rate of 1.7e-8, which is more reliable that the arbitrary estimate of 6.8e-8 obtained from an SFS based demographic analysis without fixing any population size or time divergence parameters^18^. Luckily, a rich ichthyological record of the Danube and Lake Constance documents the historical absence and only recent colonization of threespine stickleback in both regions^14,19–29^ as well as known anthropogenic introductions^14,23,29^, inconsistent with a natural postglacial colonization of Lake Constance from the Danube. Geographically highly resolved historical and contemporary data on lateral plate morph distributions in Europe^14,23,24,30–34^ further support arrival in Lake Constance from multiple sources, in line with genomic signatures of secondary contact and admixture between several lineages^2^ (Supplementary Fig. 2). Considering all evidence, a rather recent formation of a hybrid lineage in secondary contact in the Lake Constance catchment, facilitated by multiple introductions appears to be the most parsimonious scenario, in line with our earlier interpretation^2^.

### Demographic modelling of “ecological vicariance”

Berner^1^ criticized that neutral demographic models cannot adequately represent an “ecological vicariance” scenario in which divergent selection is a central component^18^. If selection-driven divergence indeed precluded inference of population history, earlier interpretations of the origin and timing of Lake Constance stickleback from a phylogeny and demographic model^1,18^ would be flawed too. But divergent selection affects genomes only locally at and around targets of selection while much of the genome evolves under neutrality, even though the extent of either might be debated^35–37^. Both empirical^18,38^ and experimental data^39,40^ in stickleback support the view that only a minority of the genome is affected by divergent selection, with the majority preserving information on the neutral history that can be harnessed in demographic modelling or phylogenetic analyses. The “ecological vicariance” hypothesis is thus certainly testable with neutral demographic models, especially because it makes a clear demographic prediction: colonization by a single lineage followed by primary divergence between ecotypes within this lineage. In our test of these neutral predictions, we were able to clearly reject an ‘ecological vicariance’ scenario over better-fitting alternative secondary contact and admixture scenarios^2^ which had not been considered previously^18^.

### Parallel evolution vs. inference of secondary contact

Berner^1^ raised an interesting possibility that parallel evolution could have biased our inference of secondary contact and admixture both in our demographic analyses and our other population genomic analyses^2^. Specifically, parallel evolution due to selection on the same alleles in similar habitats would lead to support for demographic models with admixture between independently evolved stream-adapted stickleback and signatures of excess allele sharing between such stream-adapted stickleback relative to lake-adapted stickleback closely related to one of them (Fig. 1c). In our demographic modelling, we controlled for local effects of selection by removing low recombination regions from the analysis^2^, with the rationale that effects of background and divergent selection on linked neutral variation are strongest in regions of low recombination while highly recombining regions behave mostly neutrally^36^. Berner^1^ questioned the efficacy of such a control due to the lack of a correlation between divergent selection and recombination rate in whole-genome data^40^. However, we used sparser RAD-sequencing data in our demographic modelling that does show an enrichment of divergent selection signatures in low recombination regions^18,38^ as predicted for low marker densities^41,42^. Our approach to avoid effects of selection is thus justified and should preclude effects of a possible parallel evolution bias on our demographic inference.

To confirm that our analyses of excess allele sharing are not affected by a parallel evolution bias, we now included additional stream-adapted West and South European populations that we do not expect to have contributed to the gene pool in Lake Constance. Such populations should show the same excess allele sharing with stream-adapted stickleback of Lake Constance under the parallel evolution hypothesis but not under the admixture hypothesis (Fig. 1c). We found that excess allele sharing signatures were much stronger for, or entirely confined to, Rhine and upper Rhone stickleback than for other stream populations (Fig. 1c), consistent with admixture but not with parallel evolution. Furthermore, all Lake Constance stickleback show signatures of admixture between Rhine / upper Rhone and East European lineages independent of habitat / ecotype (Supplementary Fig. 2 and Supplementary Fig. 2 in^2^), as predicted by admixture but not by a parallel ecotype evolution bias. Additionally supported by evidence for admixture from the mtDNA phylogeography^2,3^, we can confidently reject the hypothesis that parallel evolution caused false admixture signatures in Lake Constance stickleback^2^.

### Evidence for admixture-facilitation of ecological speciation

Our conclusion on admixture-facilitation of ecological speciation was deemed premature by Berner^1^, barring a more rigorous demonstration of the absence of adaptive alleles in the source populations or tracing the origin of haplotypes back to source populations. The former would be a challenging task due to uncertainty about the exact introduction or colonization routes, more recent admixture in populations along that colonization route and trade-offs between sequencing more individuals and full genomes. We welcome future research, such as haplotype-based reconstruction. Nonetheless, we believe that our evidence of divergent sorting between habitats of admixture-derived alleles in genomic regions under selection^2^ does already lend significant support to admixture-facilitation of ecological speciation.

## Methods

We added to our *SbfI* (+*PstI*) RAD-sequencing dataset^2^ previously published data from three upper Danube populations (DAN;^1^ SZO;^6^ MUR^6^), one Lake Constance stream population (GRA^1^) and one West European stream population (KIN^6^, see data availability statement below for accessions) and repeated read alignment, variant and genotype calling and filtering with the same parameters used in our previous analysis^2^. We also repeated the addition of outgroup alleles to the resulting SNP dataset, using the Black-spotted stickleback *Gasterosteus wheatlandi* genome^43^ as fixed outgroup in computations of Patterson’s D-statistic^44^ with Dsuite v0.3^45^. We used D-statistics to test (i) whether stream-adapted stickleback populations from West and South Europe, regardless of admixture history, show excess allele sharing with stream-adapted populations from Lake Constance, relative to the lake ecotype (Fig. 1c), and (ii) whether both Lake Constance and upper Danube stickleback show signatures of admixture between West and East European stickleback lineages (Supplementary Fig. 2). We used D-statistics of four taxon topologies with the highest number of shared derived alleles (‘BBAA pattern’^45^), a two-tailed standard block-jackknife procedure implemented in Dsuite with default parameters and considered p-values corrected for false discovery rate^46^ in R v4.0.2^47^ below 0.01 as significant excess allele sharing. We also repeated previous phylogenetic analyses^2^ with the three upper Danube populations and the additional Lake Constance stream population (GRA) included (Supplementary Fig. 1d), as well as for subsets excluding all Lake Constance and upper Danube populations (Supplementary Fig. 1c), including only one Lake Constance or upper Danube population or including one Lake Constance and upper Danube population each (Supplementary Fig. 1e), with filtering and parameters as used previously^2^.

### Reporting summary

Further information on research design is available in the Nature Research Reporting Summary linked to this article.

## Supplementary information

Supplementary Information

Reporting Summary

## Data Availability

Genetic data used in this study are available under the following Sequence Read Archive (SRA) accessions: DRR032274, SRX092177, SRX1555773, SRX1555774, SRX1555775, SRX1555776, SRX1555777, SRX1555778, SRX1555779, SRX1555780, SRX1555781, SRX1555782, SRX1555783, SRX1555784, SRX1555786, SRX1555799, SRX1555805, SRX1555806, SRX1555807, SRX1555808, SRX1555809, SRX1555812, SRX1555813, SRX1555814, SRX1555815, SRX1555816, SRX1555817, SRX1555818, SRX1555819, SRX1555820, SRX1555821, SRX1555822, SRX1555823, SRX1555824, SRX1555825, SRX1555826, SRX1555827, SRX1555828, SRX1555829, SRX1555830, SRX1555831, SRX1555832, SRX1555833, SRX1555834, SRX1555835, SRX1555836, SRX1555837, SRX1555838, SRX1555839, SRX1555840, SRX1555841, SRX1555842, SRX1555843, SRX1555844, SRX1555845, SRX1555846, SRX1555847, SRX1555848, SRX1555849, SRX1555850, SRX1555851, SRX1555852, SRX1555853, SRX1555854, SRX1555855, SRX1555856, SRX1555857, SRX1555858, SRX1555859, SRX1555860, SRX1555861, SRX1555862, SRX1555863, SRX1555864, SRX1555865, SRX1555866, SRX1555867, SRX1555868, SRX1555869, SRX1555870, SRX1555871, SRX1555872, SRX1555873, SRX1555874, SRX1555875, SRX1555876, SRX1555877, SRX1555878, SRX1555879, SRX1555880, SRX1555881, SRX1555882, SRX1555883, SRX1555884, SRX1555885, SRX1555886, SRX1555887, SRX1555888, SRX1555889, SRX1555890, SRX1555891, SRX1555893, SRX1555898, SRX1555899, SRX1555900, SRX1555901, SRX1555902, SRX1555903, SRX1555904, SRX1555905, SRX1555906, SRX1555907, SRX1555908, SRX1555909, SRX1555910, SRX1555911, SRX1555912, SRX1555913, SRX1555914, SRX1555915, SRX1555916, SRX1555917, SRX1555918, SRX1555919, SRX1555920, SRX1555922, SRX1555923, SRX1555924, SRX1555925, SRX1555926, SRX1555927, SRX1555928, SRX1555929, SRX1555930, SRX1555931, SRX1555932, SRX1555936, SRX1555937, SRX1555938, SRX1555939, SRX1555940, SRX1555941, SRX1555942, SRX1555943, SRX1555944, SRX1555945, SRX1555946, SRX1555947, SRX1555948, SRX3997965, SRX3997981, SRX3997982, SRX3997986, SRX3997987, SRX3997988, SRX3997989, SRX3997990, SRX3998011, SRX3998012, SRX3998013, SRX3998062, SRX3998063, SRX3998065, SRX3998066, SRX3998068, SRX3998069, SRX6084930, SRX6084931, SRX6084932, SRX6084933, SRX6084934, SRX6084935, SRX6084936, SRX6084937, SRX6084938, SRX6084939, SRX6084940, SRX6084941, SRX6084942, SRX6084947, SRX6084948, SRX6084949, SRX6084950, SRX6084951, SRX6084952, SRX6084953, SRX6084954, SRX6084955, SRX6084956, SRX6084957, SRX6084958, SRX6084959, SRX6084960, SRX6084961, SRX6084962, SRX6084963, SRX6084964, SRX6084965, SRX6084966, SRX6084967, SRX6084968, SRX6084969, SRX6084972, SRX6084973, SRX6084974, SRX6084975, SRX6084976, SRX6084977, SRX6084978, SRX6084979, SRX6084980, SRX6084981, SRX6084982, SRX6084983, SRX6084984, SRX6084985, SRX6084986, SRX6084987, SRX6084988, SRX6084989, SRX6084990, SRX6084991, SRX6084992, SRX6084993, SRX6084994, SRX6084995, SRX6084996, SRX6101711, SRX6101712, SRX6101713, SRX6101714, SRX6101715, SRX6101716, SRX6864092, SRX6864103, SRX6864114, SRX6864125. The source data underlying Fig. 1 and Supplementary Figs. 1–2 are provided as a Source Data file. Source data are provided with this paper.

## Source data

Source Data

## Code availability

All custom code used in this study has been published with our previous publication^2^.
