# Supplementary material for: Reply to “Re-evaluating the evidence for facilitation of stickleback speciation by admixture in the Lake Constance basin”
Source: Nat Commun. 2021 May 14;12:2807. doi: 10.1038/s41467-021-23096-x (PMC8121787; doi:10.1038/s41467-021-23096-x)
Supplement: Supplementary file 1 — Supplementary Information [file 41467_2021_23096_MOESM1_ESM.pdf]

Reply to "Re-evaluating the evidence for facilitation of stickleback speciation by admixture in the Lake Constance basin"

Marques et al.

Supplementary Figures

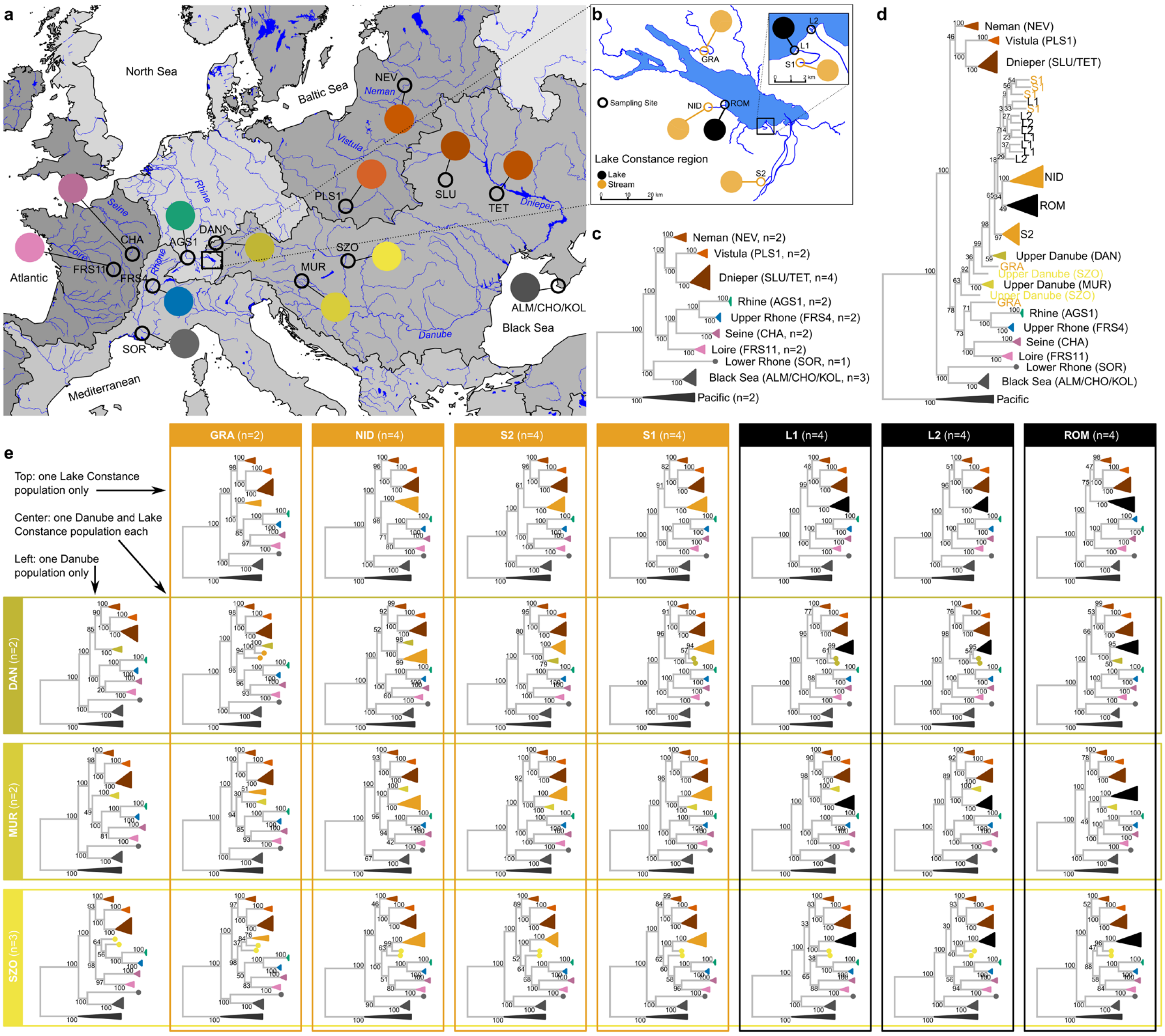

Supplementary Figure 1 | Topology and clade support change with the inclusion of hybrid populations. Lake Constance and upper Danube populations cluster with either West or East European stickleback lineages in phylogenetic analyses involving fewer hybrid populations, but form a monophyletic, intermediately placed hybrid clade in a phylogenetic analysis containing all hybrid populations (except the Lake Constance population GRA, see text). (a) Map of European stickleback populations used in phylogenetic analyses with colors indicating population identity, with (b) inset showing the Lake Constance region where colors indicate lake or stream habitat. (c) Maximum likelihood phylogeny of European stickleback excluding Lake Constance and Danube hybrid populations. Triangles indicate multiple collapsed tips in the tree, a point a single sample. (d) Maximum likelihood phylogeny of major lineages of European stickleback including all Lake Constance and Danube hybrid populations clusters them with West European stickleback with modest bootstrap support. (e) Maximum likelihood phylogenies of major lineages of European stickleback including only one upper Danube population (left column, yellow hues), only one Lake Constance population (top row, orange color) or one upper Danube

and one Lake Constance population at a time (center phylogenies, black). The frames indicate which upper Danube and Lake Constance population is included in each phylogeny. Context-dependent topologies and reduced internal bootstrap support on nodes connecting the putative parental lineages are consistent with a hybrid origin[1] of Lake Constance and upper Danube stickleback. Branch labels indicate bootstrap support. Triangles indicate multiple collapsed tips in the tree, a point a single sample. Watershed maps are derived from “Water Base: Global River Basins” by The World Bank used under CC BY 4.0, river and lake maps from “European catchments and Rivers network system (Ecrins)” by the European Environment Agency (EEA). Source data are provided as a Source Data file.

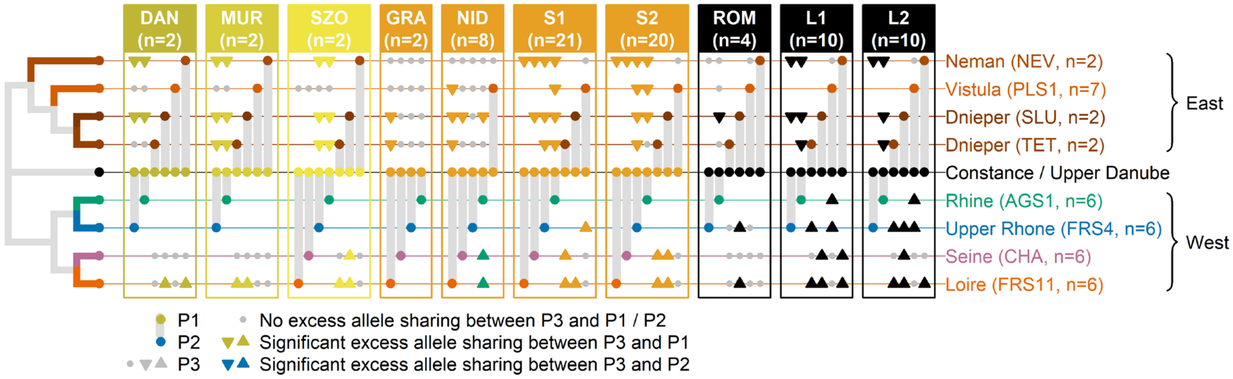

Supplementary Fig. 2 | Evidence for an admixed origin of upper Danube and Lake Constance stickleback. As sisters of West European stickleback (Rhine, upper Rhone, Seine, Loire), all upper Danube stickleback and Lake Constance populations show excess allele sharing with at least one East European stickleback population (NEV, PLS1, SLU, TET). Grey vertical bars indicate P1 / P2 sister relationships in 4-taxon topologies with *Gasterosteus wheatlandi* as outgroup (not shown in the tree), which are supported by the highest shared derived allele count (number of "BBAA" patterns) among the three P1 / P2 / P3 trio topologies. Grey dots indicate P3 populations without excess allele sharing with either P1 or P2, while triangles indicate P3 populations with significant excess allele sharing with either P1 or P2 (D_{BBAA}>0 or D_{BBAA}<0, two-tailed standard block-jackknife procedure, false discovery rate adjusted $p < 0.01$). Colors of dots and triangles correspond to population identity indicated on top and on the right of the figure. Only P1 / P2 sister relationships between an upper Danube or Lake Constance and one of the other eight populations are shown. Source data are provided as a Source Data file.

Supplementary References

1. Seehausen, O. Hybridization and adaptive radiation. *Trends Ecol Evol* **19**, 198–207 (2004).
