# Supplementary material for: Reply to “Re-evaluating the evidence for facilitation of stickleback speciation by admixture in the Lake Constance basin”
Source: Nat Commun. 2021 May 14;12:2807. doi: 10.1038/s41467-021-23096-x (PMC8121787; doi:10.1038/s41467-021-23096-x)
Supplement: Supplementary file 2 — Reporting Summary [file 41467_2021_23096_MOESM2_ESM.pdf]

nature research

Corresponding author(s): Ole Seehausen

Last updated by author(s): Apr 14, 2021

Reporting Summary

Nature Research wishes to improve the reproducibility of the work that we publish. This form provides structure for consistency and transparency in reporting. For further information on Nature Research policies, see our Editorial Policies and the Editorial Policy Checklist.

Statistics

For all statistical analyses, confirm that the following items are present in the figure legend, table legend, main text, or Methods section.

n/a	Confirmed	
[ ]	[x]	The exact sample size (*n*) for each experimental group/condition, given as a discrete number and unit of measurement
[ ]	[x]	A statement on whether measurements were taken from distinct samples or whether the same sample was measured repeatedly
[ ]	[x]	The statistical test(s) used AND whether they are one- or two-sided<br>*Only common tests should be described solely by name; describe more complex techniques in the Methods section.*
[x]	[ ]	A description of all covariates tested
[ ]	[x]	A description of any assumptions or corrections, such as tests of normality and adjustment for multiple comparisons
[ ]	[x]	A full description of the statistical parameters including central tendency (e.g. means) or other basic estimates (e.g. regression coefficient) AND variation (e.g. standard deviation) or associated estimates of uncertainty (e.g. confidence intervals)
[ ]	[x]	For null hypothesis testing, the test statistic (e.g. *F*, *t*, *r*) with confidence intervals, effect sizes, degrees of freedom and *P* value noted<br>*Give P values as exact values whenever suitable.*
[x]	[ ]	For Bayesian analysis, information on the choice of priors and Markov chain Monte Carlo settings
[x]	[ ]	For hierarchical and complex designs, identification of the appropriate level for tests and full reporting of outcomes
[x]	[ ]	Estimates of effect sizes (e.g. Cohen's *d*, Pearson's *r*), indicating how they were calculated

Our web collection on statistics for biologists contains articles on many of the points above.

Software and code

Policy information about availability of computer code

Data collection	No software was used for data collection.
Data analysis	BOWTIE 2 v2.0.0 for read alignment, GATK v2.770 BASERECALIBRATOR and UNIFIEDGENOTYPER for calibration, variant and genotype calling, BCFTOOLS v1.871 and VCFTOOLS v0.1.12b72 for variant and genotype filtering, RAXML 8.0.2673 for phylogenetic analysis, DSUITE v0.3 for Dstats calculation, R v4.0.2 for computation of false discovery rate correction and plotting data, QGIS v3.8.1 for map creation, INKSCAPE v0.92.4 for map and figure merging.

For manuscripts utilizing custom algorithms or software that are central to the research but not yet described in published literature, software must be made available to editors and reviewers. We strongly encourage code deposition in a community repository (e.g. GitHub). See the Nature Research guidelines for submitting code & software for further information.

Data

Policy information about availability of data

All manuscripts must include a data availability statement. This statement should provide the following information, where applicable:
- Accession codes, unique identifiers, or web links for publicly available datasets
- A list of figures that have associated raw data
- A description of any restrictions on data availability

Genetic data used in this study are available under the following accessions: DRR032274, SRX092177, SRX1555773, SRX1555774, SRX1555775, SRX1555776, SRX1555777, SRX1555778, SRX1555779, SRX1555780, SRX1555781, SRX1555782, SRX1555783, SRX1555784, SRX1555786, SRX1555799, SRX1555805, SRX1555806, SRX1555807, SRX1555808, SRX1555809, SRX1555812, SRX1555813, SRX1555814, SRX1555815, SRX1555816, SRX1555817, SRX1555818, SRX1555819, SRX1555820, SRX1555821, SRX1555822, SRX1555823, SRX1555824, SRX1555825, SRX1555826, SRX1555827, SRX1555828, SRX1555829, SRX1555830, SRX1555831, SRX1555832, SRX1555833, SRX1555834, SRX1555835, SRX1555836, SRX1555837, SRX1555838, SRX1555839, SRX1555840, SRX1555841, SRX1555842, SRX1555843, SRX1555844,

April 2020

SRX1555845, SRX1555846, SRX1555847, SRX1555848, SRX1555849, SRX1555850, SRX1555851, SRX1555852, SRX1555853, SRX1555854, SRX1555855, SRX1555856, SRX1555857, SRX1555858, SRX1555859, SRX1555860, SRX1555861, SRX1555862, SRX1555863, SRX1555864, SRX1555865, SRX1555866, SRX1555867, SRX1555868, SRX1555869, SRX1555870, SRX1555871, SRX1555872, SRX1555873, SRX1555874, SRX1555875, SRX1555876, SRX1555877, SRX1555878, SRX1555879, SRX1555880, SRX1555881, SRX1555882, SRX1555883, SRX1555884, SRX1555885, SRX1555886, SRX1555887, SRX1555888, SRX1555889, SRX1555890, SRX1555891, SRX1555893, SRX1555898, SRX1555899, SRX1555900, SRX1555901, SRX1555902, SRX1555903, SRX1555904, SRX1555905, SRX1555906, SRX1555907, SRX1555908, SRX1555909, SRX1555910, SRX1555911, SRX1555912, SRX1555913, SRX1555914, SRX1555915, SRX1555916, SRX1555917, SRX1555918, SRX1555919, SRX1555920, SRX1555922, SRX1555923, SRX1555924, SRX1555925, SRX1555926, SRX1555927, SRX1555928, SRX1555929, SRX1555930, SRX1555931, SRX1555932, SRX1555936, SRX1555937, SRX1555938, SRX1555939, SRX1555940, SRX1555941, SRX1555942, SRX1555943, SRX1555944, SRX1555945, SRX1555946, SRX1555947, SRX1555948, SRX3997965, SRX3997981, SRX3997982, SRX3997986, SRX3997987, SRX3997988, SRX3997989, SRX3997990, SRX3998011, SRX3998012, SRX3998013, SRX3998062, SRX3998063, SRX3998065, SRX3998066, SRX3998068, SRX3998069, SRX6084930, SRX6084931, SRX6084932, SRX6084933, SRX6084934, SRX6084935, SRX6084936, SRX6084937, SRX6084938, SRX6084939, SRX6084940, SRX6084941, SRX6084942, SRX6084947, SRX6084948, SRX6084949, SRX6084950, SRX6084951, SRX6084952, SRX6084953, SRX6084954, SRX6084955, SRX6084956, SRX6084957, SRX6084958, SRX6084959, SRX6084960, SRX6084961, SRX6084962, SRX6084963, SRX6084964, SRX6084965, SRX6084966, SRX6084967, SRX6084968, SRX6084969, SRX6084972, SRX6084973, SRX6084974, SRX6084975, SRX6084976, SRX6084977, SRX6084978, SRX6084979, SRX6084980, SRX6084981, SRX6084982, SRX6084983, SRX6084984, SRX6084985, SRX6084986, SRX6084987, SRX6084988, SRX6084989, SRX6084990, SRX6084991, SRX6084992, SRX6084993, SRX6084994, SRX6084995, SRX6084996, SRX6101711, SRX6101712, SRX6101713, SRX6101714, SRX6101715, SRX6101716, SRX6864092, SRX6864103, SRX6864114, SRX6864125. The source data underlying Fig. 1 and Supplementary Figs. 1-2 are provided as a Source Data file.

Field-specific reporting

Please select the one below that is the best fit for your research. If you are not sure, read the appropriate sections before making your selection.

☐ Life sciences ☐ Behavioural & social sciences ☒ Ecological, evolutionary & environmental sciences

For a reference copy of the document with all sections, see nature.com/documents/nr-reporting-summary-flat.pdf

Ecological, evolutionary & environmental sciences study design

All studies must disclose on these points even when the disclosure is negative.

Study description	Biogeographic reconstruction of the origin and evolution of Lake Constance stickleback, reply to Matters Arising manuscript.
Research sample	Threespine stickleback (Gasterosteus aculeatus), broad biogeographic sampling of watersheds surrounding the geographic area around Lake Constance including catchments draining into the Atlantic, Black Sea, Baltic Sea, North Sea and Mediterranean.
Sampling strategy	Use of previously published datasets with at least 1 individual per population for genomic analyses.
Data collection	No new data collected for this publication.
Timing and spatial scale	We used previously published data from 21 threespine stickleback freshwater population in West, Central, South and East Europe, two freshwater populations in the East Pacific and from one blackspotted stickleback Gasterosteus wheatlandi. According to these previous studies, fish were collected between 2003 and 2015.
Data exclusions	No data was excluded.
Reproducibility	No attempts to repeat the experiment have been conducted yet.
Randomization	Not relevant for this study, no randomization was performed.
Blinding	Blinding was not relevant for our genomic study, given that no observer-biased measurements were taken.

Did the study involve field work? ☐ Yes ☒ No

Reporting for specific materials, systems and methods

We require information from authors about some types of materials, experimental systems and methods used in many studies. Here, indicate whether each material, system or method listed is relevant to your study. If you are not sure if a list item applies to your research, read the appropriate section before selecting a response.

Materials & experimental systems

n/a	Involved in the study
☒	☐ Antibodies
☒	☐ Eukaryotic cell lines
☒	☐ Palaeontology and archaeology
☒	☐ Animals and other organisms
☒	☐ Human research participants
☒	☐ Clinical data
☒	☐ Dual use research of concern

Methods

n/a	Involved in the study
☒	☐ ChIP-seq
☒	☐ Flow cytometry
☒	☐ MRI-based neuroimaging
